# Nature of the evidence base and strengths, challenges and recommendations in the area of nutrition and health claims: a position paper from the Academy of Nutrition Sciences

**DOI:** 10.1017/S0007114522003300

**Published:** 2023-07-28

**Authors:** Margaret Ashwell, Mary Hickson, Sara Stanner, Ann Prentice, Christine M. Williams

**Affiliations:** 1 Trustees, The Academy of Nutrition Sciences, London, W6 7NJ, UK; 2 British Dietetic Association, Interchange Place, Birmingham, UK; 3 British Nutrition Foundation, London, UK; 4 MRC Nutrition and Bone Health Group, Cambridge, UK

**Keywords:** Health claims, Evidence base, Consumers, Health professionals

## Abstract

The regulation of health claims for foods by the Nutrition and Health Claims Regulation is intended, primarily, to protect consumers from unscrupulous claims by ensuring claims are accurate and substantiated with high quality scientific evidence. In this position paper, the Academy of Nutrition Sciences uniquely recognises the *strengths* of the transparent, rigorous scientific assessment by independent scientists of the evidence underpinning claims in Europe, an approach now independently adopted in UK. Further *strengths* are the separation of risk assessment from risk management, and the extensive guidance for those submitting claims. Nevertheless, four main *challenges* in assessing the scientific evidence and context remain: (i) defining a healthy population, (ii) undertaking efficacy trials for foods, (iii) developing clearly defined biomarkers for some trial outcomes and (iv) ensuring the composition of a food bearing a health claim is consistent with generally accepted nutrition principles. Although the Regulation aims to protect the consumer from harm, we identify some *challenges* from consumer research: (i) making the wording of some health claims more easily understood and (ii) understanding the implications of the misperceptions around products bearing nutrition or health claims. *Recommendations* are made to overcome these challenges. Further, the Academy *recommends* that a dialogue is developed with the relevant national bodies about Article 12(c) in the Regulation. This should further clarify the GB Guidance to avoid the current non-level playing field between health professionals and untrained ‘influencers’ who are not covered by this Article about the communication of authorised claims within commercial communications.

This position paper is the second in a series from the Academy of Nutrition Sciences. The first paper considered the nature of the science evidence base and frameworks underpinning dietary recommendations for prevention of non-communicable diseases^(1)^. In this second paper, we discuss the EU Nutrition and Health Claims Regulation (‘the Regulation’) which was first published in 2006^(2)^. This paper provides a summary of the Regulations for the EU and UK, with reference to approaches used by other countries for comparison. We refer to the learnings that have been gained through the implementation of the European Food Safety Authority (EFSA) evidence-based process for assessment of proposed claims, provide examples of different types of authorised claims, discuss potential challenges for those who submit or assess claims for approval, as well as concerns arising from consumer research and from health professionals in their interpretation of the official GB Guidance on Health Claims^(3)^. The paper is not a comprehensive or systematic review of the Health Claims literature; there are a number of recently published, authoritative reviews available for those seeking deeper insight into this complex area of Regulation^(4,5,6)^. Our aim is to enable non-specialists to appreciate the progress that has been made in recent years and to focus on the remaining challenges for the science, the consumer and for key stakeholders, including practising health professionals. We hope this will open up a discourse between the Regulatory Authorities and organisations who represent these key stakeholders. The latter includes the Academy of Nutrition Sciences (www.academyofnutritionsciences.org), whose member organisations include scientific societies, professional bodies and a nutrition information charity.

## Regulatory frameworks and types of health claims

The introduction of nutrition labelling in the EU in 1990^(7)^ was intended to provide nutrition information for the public and help inform consumer choice. The labelling legislation at that time was for voluntary nutrient declarations, but these had to comply with a particular format, i.e., values for energy, protein, carbohydrate, fat, fibre, sodium, and, in certain circumstances, vitamins and minerals as long as a minimum level was present in the food. This led to provision of a wealth of voluntary nutrition information on food products and, over time, provision of nutrition information in a specified format became a legal requirement in certain contexts, such as when an authorised claim was made for a particular nutrient contained in the food, or if a food was fortified. This legislation has subsequently been updated and revised.^(8)^.

The EU Nutrition and Health Claims Regulation was first published in 2006^(2)^ and extended later. A number of significant EC projects funded in the preceding decade, such as FUFOSE^(9)^ and PASSCLAIM^(10)^ had addressed the issues of scientific assessment of health claims and thus paved the way for the Regulation. Under the auspices of the Regulation, information on a food label about the nutritional content and health effects of the food is intended to provide guidance for consumers and to act as an incentive for the food industry to develop and market healthier food products, as well as providing a level playing field for industry with common rules to ensure fair competition. Importantly, to protect the public, the Regulation requires there to be robust scientific evidence to support any claims made (explicit as well as implicit), ensuring that foods are not selected and consumed as a direct result of misleading claims about their purported beneficial effects. Procedures for assessing the available evidence are in place (see ‘The nature of the evidence base underpinning health claims in the EU and UK’ section).

The Regulation applies to all nutrition and health claims made in commercial communications, whether in the labelling, presentation or advertising of foods to be delivered as such to the final consumer. The Regulation does not apply to claims that are made in non-commercial communications, such as dietary guidelines or advice issued by public health authorities and bodies, or non-commercial communications and information in the press and in scientific publications. Detailed guidance is available on the interpretation of ‘commercial communications’ and on the implications for individual health professionals^(11)^ (see ‘Health professionals and health claims’ section).

The 2006 Regulation includes a list of permitted nutrition claims such as high fibre, low fat, sugar free, source of vitamins and minerals and their conditions of use. However, the Regulation mainly focuses on health claims. The definition of a health claim (Article 2 in the Regulation) is ‘any claim that states, suggests or implies that a relationship exists between a food category, a food or one of its constituents and health’. It introduced the possibility for food producers and retailers to make claims which related a component in the food product to a physiological benefit or a reduction in disease risk (see ‘The nature of the evidence base underpinning health claims in the EU and UK’ section), based on the outcome of an EU-wide process focussed on a thorough, independent assessment of the scientific evidence.

Health claims for foods are intended as an important communication tool to inform and direct consumer choice, and procedures for regulating the validity and use of such claims are in place in many countries and jurisdictions worldwide. Procedures were implemented in Japan in the 1980s, in USA in the 1990s, in UK and EU in the early 2000s and in Australia and New Zealand in the later 2000s (see ‘Types of health claims in other countries – how do they differ from EU and UK?’ section). These have continued to evolve and develop as the science, and the regulatory processes, have improved^(4)^. Although approaches to the scientific assessment of health claims are similar, there are some between-country differences in regulatory frameworks and processes, which reflect their stage of development, as well as cultural and political factors^(6)^. Some of these differences will be briefly discussed in the following sections, especially in relation to how procedures in other countries differ from those now used in the EU and UK.

## Background to health claims in EU and UK

Since the EU legislation for regulating health claims was published in 2006^(2)^, the guidance for submitting health claim applications has evolved. An EU register of approved claims has been developed^(12)^ and, over time, EFSA has gained valuable insight into the challenges faced by expert committees responsible for evaluating the scientific evidence, and by policy makers in formulating authoritative consumer guidance on the potential health benefits of foods, nutrients, and other food components^(13)^. This insight has been used to update the guidance for those preparing the detailed scientific dossiers required as support for health claims applications on behalf of food businesses. Updates on the status of health claims in Europe have been provided at regular intervals^(14,15,5)^.

The UK previously had its own system of assessing health claims which was launched in 2000^(16)^. However, since 2006, and until the UK left the EU at the end of 2020, health claims made in the UK were regulated by the European Commission^(2)^. In January 2021, a new committee, the UK Nutrition and Health Claims Committee (UKNHCC), took over responsibility for the assessment of the scientific evidence in support of submitted new claims^(17)^ in Great Britain. Claims made in Northern Ireland (the fourth country in the UK along with England, Scotland and Wales) still fall under the auspices of the EU. The UKNHCC operates in a similar way to EFSA and intends to have similar timescales. It also uses an evidence evaluation process similar to that used by EFSA and to the processes used by the UK Scientific Advisory Committee on Nutrition, described in the first position paper from the Academy^(1)^. At the beginning of 2021, the EU Register of authorised claims was adopted by the UK for use in Great Britain^(3)^. As the new UK committee has started its work, with an assessment process that is now separate to that followed by the EU, there is the potential for divergence in submitted and approved claims.

## Types of health claims in the European Union and UK

In the 2006 EU legislation^(2)^, now adopted by the UK^(3)^, three main types of health claims are identified as described below. Submissions under Article 13(1) are no longer open, but can instead be made via the process described in Article 13(5):

- Function claims (Articles 13(1) and 13(5))
- Reduction of disease risk claims (Article 14(1)(a))
- Health claims referring to children’s development (Article 14(1)(b))

### Article 13 function claims

These cover the role of nutrients (or other food substances) on physiological function and fall into one of three sub-types: (i) growth, development and the functions of the body; (ii) psychological and behavioural function and (iii) slimming or weight control, or a reduction in the sense of hunger, or an increase in the sense of satiety, or the reduction in available energy from the diet.

*Article 13(1) claims* are those based on well-established roles of foods and essential nutrients, sometimes referred to as ‘textbook’ claims, and the extent of the supporting evidence required was proportionate. The vast majority of the submitted claims that achieved authorisation were for micronutrients and were based on their well-established role as essential nutrients. Following the initial 2006 call for submission of candidate function claims, the EC consolidated a very large list and the EFSA Panel on Nutrition, Dietetic Products and Allergies (the NDA panel, composed of independent scientists) reviewed over half the claims drawn from this list. The complete list was published on the EFSA website in the form of an Access database in May 2010 and is now continuously updated and available via the EC’s website^(12)^ showing whether the opinion reached by the assessors was positive or negative, whether it has since been authorised by the Commission, and any conditions of use. The authorised claims with their conditions of use were adopted into law in 2012, and several revisions have been produced since then^(18)^. Two examples of Article 13(1) claims are

‘Vitamin C contributes to the normal function of the immune system’^(19)^

‘Calcium and vitamin D are needed for the maintenance of normal bone’^(20)^

Conditions of use apply to these claims. Manufacturers can only use these claims if the food is a ‘source’ of the nutrient, legally defined as containing at least 15 % of the nutrient reference value in 100 g of the food^(7)^.

Although most of the authorised claims in the initial list published in 2010 concerned the well-established function of an essential nutrient, some were authorised on the basis of evidence for a physiological effect associated with consumption of the food. The scientific evidence for these was scrutinised using the full EFSA process, i.e. scientifically substantiated by taking into account the totality of the available scientific data and by weighing the evidence (see ‘The nature of the evidence base underpinning health claims in the EU and UK’ section). Two examples of such authorised claims are as follows:

Replacing digestible starches with resistant starch in a meal contributes to a reduction in the blood glucose rise after that meal. The conditions of use state that ‘The claim may be used only for food in which digestible starch has been replaced by resistant starch so that the final content of resistant starch is at least 14 % of total starch’^(21)^.

Carbohydrate-electrolyte solutions contribute to the maintenance of endurance performance during prolonged endurance exercise (specific compositional criteria apply)^(22)^.

There are still over 2000 unresolved applications, concerning botanical claims, remaining from the original tranche submitted to EFSA in response to the first call in 2006. These are awaiting either an EFSA risk assessment or Commission approval following the EFSA opinion. The main issue for these applications is the paucity of high-quality human studies and the question of whether to permit ‘traditional use’ evidence to support such claims. These claims remain ‘on-hold’ at the risk management level while it is decided how they should be evaluated^(23,5)^.

*Article 13(5) health claims* are based on newly developed scientific evidence and/or applications which include a request for the protection of proprietary data. These claims are scientifically assessed by considering the totality of the available scientific data and by weighing the evidence (the full EFSA process that requires submission of a detailed dossier of evidence in support of the claim). This is now the only application route for new function claims. Here are two examples of Article 13(5) claims:

*‘Sugar beet fibre contributes to an increase in faecal bulk’.* This claim can only be used on a food which is defined as ‘high’ in that fibre as defined in legislation^(24)^.

‘*Daily creatine consumption can enhance the effect of resistance training on muscle strength in adults over the age of 55’.* The use of this claim is dependent on the food meeting specific criteria^(25)^.

The version of the register of claims that existed at the point when the UK left the EU at the beginning of 2021 has been adopted for use in Great Britain.

### Article 14(1)(a) reduction of disease risk claims

This Article is intended for any health claim that states, suggests or implies that the consumption of a food category, a food or one of its constituents significantly reduces a risk factor implicated in the development of a human disease. Importantly, such health claims must address reduction in *risk factor(s) for a* disease, not a reduction in the disease itself (which would be covered by the Medicines and Medical Devices Act^(26)^. This evidence, that the food or nutrient reduces a risk factor for disease, has implications for the scientific assessment of the claim, since the relationship between apparent risk factors for any disease and the disease itself is not always very strong, or there may be no, as yet, identifiable risk factors. This can make substantiation and assessment of Article 14(1) claims somewhat challenging.

To time of writing, fourteen claims under Article 14(1)(a) have been authorised in the EU, and 27 applications have not received authorisation (see Table 1).

**Table 1.** Summary of health claims evaluated under European nutrition and health claims regulation 2006–2020

Claim type:	Evaluated by EFSA panel	Positive opinion	Negative opinion	% Applications authorised
Function, Article 13(1)†	2758	229*	1875	N/A
Function, Article 13(5)	142	12	130	8 %
Reduction of disease risk reduction, Article 14(1)(a)	41	14	27	34 %
Children’s development, Article 14(1)(b)	57	12	45	21 %

EFSA, European Food Safety Authority.

* May include duplicate claims made under single opinions N/A; not applicable due to differences in approval process used between Article 13(1) claims and the other categories.

† New claims no longer allowed.

One of the earliest Article 14(1)(a) claims to be authorised was for plant sterol and stanol esters. The exact wording of the claim is:

‘Plant sterols and plant stanol esters have been shown to lower/reduce blood cholesterol. High cholesterol is a risk factor in the development of coronary heart disease’^(27)^. Disclaimer added later that such products are not intended for people who do not need to control their blood cholesterol level^(28)^.

It is noteworthy that Article 14 claims contain two parts, both of which must be included when the claim is used, with the second sentence referring to the relationship between the risk factor and disease. There are also other conditions of use. In order to bear the claim, any communication about the food, most often the food label, must also state that the beneficial effect, in this case, is obtained with a daily intake of a defined amount of plant sterols or stanols.

Another example of an Article 14(1)(a) claim is for unsaturated fatty acids:

‘Consumption of saturated fat increases blood cholesterol concentrations; consumption of mono- and/or polyunsaturated fat in replacement of saturated fat has been shown to lower/reduce blood cholesterol. Blood cholesterol lowering may reduce the risk of (coronary) heart disease’.^(29)^.

In order to bear the claim, significant amounts of mixed SFA should be replaced by cis-MUFA and/or cis-PUFA in foods or diets on a gram-per-gram basis. The target population is individuals who want to lower their blood cholesterol. Again, there are conditions of use; the claim may be used only for food that is high in unsaturated fatty acids (see ‘The nature of the evidence base underpinning health claims in the EU and UK’ section)^(7)^

### Article 14(1)(b) claims referring to children’s development and health

Claims relating to children’s health are another challenging area. To date, twelve applications under Article 14(1)(b) have been authorised in the EU and 45 have not received authorisation (see Table 1). There are also approximately 25 children’s claims that have received positive EFSA opinions, but have not yet been through the EC’s authorisation process and so are considered ‘on-hold’. The EC requested comments in January 2018 on a draft proposal that these claims should only be authorised when they relate to nutrients that are not already added on a mandatory basis. Alternative options discussed were to prohibit all health claims on foods for infants and young children, which may be difficult to legislate for, or to allow health claims for both optional and mandatory nutrients. This discussion is ongoing, and the issue has not yet been resolved. It is worth noting that, should these claims achieve authorisation via the European Commission, as with any claims approved post December 2020, they will not automatically be added to the approved list of claims for Great Britain^(30)^

One of the earliest Article14(1)(b) claims to be authorised was for Ca and bone. The exact wording of the claim is as follows:

*‘Calcium is needed for normal growth and development of bone in children’.* The claim can be used only for food which is at least a ‘source’ of calcium as referred to in the claim, with the source defined in EC legislation^(31)^.

A more recently approved children’s’ claim is the following:

*‘Vitamin D contributes to the normal function of the immune system in children’* ^(32)^. The claim may be used only for food which is at least a source of vitamin D as referred to in the claim as a ‘source’^(7)^

Table 1 shows a summary of the numbers of health claims that have been evaluated and given positive or negative opinions by EFSA since 2006 under these categories. It should be noted that the attainment of a positive claim does not ensure that a claim will be authorised as the latter is the responsibility of the risk managers and the EC. Further details on the separation of risk assessment and risk management are discussed in greater detail in ‘The strengths and challenges of the health claims process followed in the EU and UK’ section.

The low rate of positive opinions for Article 13(1) function claims almost certainly represents the exceptionally large number of submissions made under this Article when the first call was made under the new legislation in 2006. Many of these were duplicates and were dealt with by combining submissions in a single opinion. New submissions for these types of claims are no longer possible. All submissions must now be made under Articles 13(5), 14(1)(a) or 14(1)(b). Table 1 shows that, since the response to the initial call in 2006, a relatively modest number of submissions has been made to date (2022). Improved guidance developed in recent years has improved the quality, but reduced the number of submissions made, achieving reasonable levels of success for those submissions.

## The nature of the evidence base underpinning health claims in the EU and UK

### Principles for scientific substantiation

Dossiers submitted in the EU and UK as an application for a new health claim undergo a full scientific assessment by the relevant expert panel (EFSA NDA Panel or UKNHCC), taking account of the totality of the available scientific data, the characterisation of the food or constituent and weighing the evidence provided in the applicant’s dossier and other materials^(33)^.The substantiation of health claims requires that the evidence fulfils three specific principles. These are shown in Table 2. Further, the claimed effect of the food or constituent needs to be reliably repeated in the target population and under the same conditions of use. The rationale underlying these principles is that the effects claimed can be achieved in the significant majority of the target population, thereby ensuring reasonable confidence in the claim for a consumer purchasing a product displaying an authorised health claim.

**Table 2.** Principles for scientific substantiation of a health claim

1. The food or constituent must be fully characterised†
2. The claimed effect is essentiality of a nutrient, OR Is a measurable, beneficial physiological effect *in vivo* in humans*
3. A cause–effect relationship exists between the food or constituent and the claimed effect

* For the target population and under the proposed conditions of use.

† This is one of the main reasons why ‘natural’ foodstuffs such as fruit/vegetables have so few authorised claims.

### The evidence base

The primary evidence used to fulfil these requirements and substantiate submitted health claims is based on human efficacy studies carried out with the food or constituent included in the proposed claim and with study groups representative of the target population. The outcome measure(s) needs to be appropriate for the physiological health effect being claimed and under the proposed conditions of use for the claim. In particular, a function claim cannot refer to a disease outcome, as mentioned before^(26)^. A disease risk reduction claim needs to refer to the reduction of a risk factor for a specific disease, not to prevention/treatment of the disease itself.

The design and conduct of the human studies should be of the highest quality with low risk of bias and confounding (i.e. where something other than the factor being studied could be causing the results seen in the study). Systematic reviews and meta-analyses of efficacy trials may be of value to the expert assessment panels in determining the overall findings for a particular food or constituent. These can help determine the overall size of the effect, its consistency or dose–response from summated findings from a number of different studies. These analyses contribute valuable evidence for a cause–effect relationship between the food or constituent and its impact on health. However, the expert panels will interrogate the data from the individual studies as well as the summated findings. Flaws in trial design, conduct, analysis and/or reporting can result in over- or underestimating the effects of an individual efficacy trial. Assessing the quality of each trial (e.g. low risk of bias in the reported results) is therefore a critical step for assessors when weighing the evidence. These, and other considerations, are important challenges for the expert panels when evaluating the evidence provided for any health claim application. Many of these challenges are covered by existing tools such as the Cochrane risk-of-bias tool for randomised controlled trials^(34)^.

The criteria for causality can be achieved through human trials but, as discussed in our first position paper, establishing biological plausibility often requires the use of model systems^(1)^. These types of studies are referred to as ‘supportive studies’, which include efficacy studies in animals as well as mechanistic studies in animals, cells and humans. Taken together, efficacy trials in humans and mechanistic studies form the evidence used to substantiate new health claims.

Although observational studies, such as high-quality prospective cohort studies, provide the core evidence used to develop dietary guidance for whole populations, these are insufficient for substantiating a health claim for a specific food or constituent. The data they provide are not able to fulfil the three key principles outlined in Table 2 ^(1)^. The level of precision in observational studies for estimates of dietary, nutrient and food intakes and the ability to adequately characterise a specific food or constituent in population studies are low. They are not able to study effects of essential nutrients with sufficient precision to support cause and effect relationships, examine direct physiological health effects in humans or investigate potential mechanisms underlying effects of foods or constituents. The main value of cohort studies with respect to health claims would be to generate valid hypotheses about potential relationships between foods or food constituents and health. These can then be further investigated to determine the likely active component of the food that is responsible for the observed health effect, thereby leading to a potential health claim.

Efficacy trials of foods can be considered equivalent to clinical drug trials in terms of rigour and repeatability, by giving the required level of confidence and protection to consumers. Nevertheless, food efficacy trials bring considerable challenge to investigators and volunteers, and some of these are discussed further in ‘The strengths and challenges of the health claims process followed in the EU and UK’ section. A more fundamental challenge is whether an assessment approach which focuses on the impact of a single ingredient or food, using a pharmaceutical model, will continue to be relevant given developments which emphasise the importance of a focus on the healthiness of whole diets^(5,35)^. Debate concerning the classification and nutritional benefits of so-called ultra-processed foods^(36)^ and developments that provide ranked scores for individual foods based on their holistic health effects^(37)^ are likely to influence scientific debate and consumer perceptions concerning health claims on foods for the foreseeable future.

### The scientific assessment process

The expert panel (EFSA, or UKNHCC for claims submitted for use in Great Britain) assesses the submitted dossier of evidence to determine the quality and consistency of the human efficacy trials, and whether studies are supportive that the three principles in Table 2 have been met. The assessment has three basic steps: (1) assembling the data into lines of evidence of similar type, (2) weighing the quality of the evidence (reliability, relevance and consistency) and (3) integrating the evidence from different types of studies. This systematic framework of assessment is similar to that used by other bodies who need to assess the scientific evidence concerning food and health^(1)^.

Following assessment, EFSA (and now UKNHCC) issues one of three categories of opinion: positive, failure to demonstrate cause and effect or insufficient evidence. The assessment panel’s opinion is then passed to the risk managers (the EC for the EU, the UK Four Nation Group for the UK^(38)^) for the second part of the assessment process (see ‘The nature of the evidence base underpinning health claims in the EU and UK’ section). Occasionally, positive EFSA opinions have not resulted in authorised health claims, or have been changed by the risk managers (see ‘The nature of the evidence base underpinning health claims in the EU and UK’ section).

EFSA has recently introduced an additional step into the process of weighing the evidence, namely the assessment of uncertainty surrounding the evidence^(39)^. It is not yet clear how this assessment of uncertainty will be managed during EFSA’s assessment process, nor how it will impact on the wording of a panel opinion or of a health claim. Assessment of uncertainty is an important step which acknowledges the complex nature of the evidence base and dependence upon human judgements which may differ (and potentially cause confusion among consumers).

Insufficient characterisation of a food or constituent is one of the reasons that a health claim submission can fail. Prominent examples have involved submitted claims for probiotic foods where the microorganism profile within a product had not been sufficiently characterised^(40)^. Claims for health effects for dietary fibre have also failed to be substantiated either due to inability to characterise the active constituent in the particular dietary fibre or because the physiological effect(s) claimed have not been sufficiently characterised or demonstrated to be linked directly with the product^(41)^. However, claims where the specific active fibre constituent has been characterised have received favourable opinions:

Consumption of arabinoxylan as part of a meal contributes to a reduction of the blood glucose rise after that meal^(42)^.

Vague or non-specific terms for physiological outcomes such as ‘gut health’, ‘healthy microbiota’, ‘natural defences’ or ‘reduction of inflammation’ are not considered sufficiently defined or measurable to justify health claims. Reliance on studies of effects of food or constituents on subjects with disease conditions will also result in an unfavourable opinion as the Regulation requires that any effects are demonstrated with healthy subjects^(13)^.

## Types of health claims in other countries: how do they differ from EU and UK?

Although this position paper is primarily about the health claims process in the EU and UK, we have briefly summarised and compared the processes used in USA, Japan, Australia and New Zealand in order to help identify the strengths and challenges of the systems and whether a greater variety of types of health claims might be beneficial for the EU and UK. For clarity, comparative examples are given for the wording of different types of claims and their conditions, for EU/UK and other countries systems. A more detailed discussion for other countries’ approaches (except Japan) can be found in elsewhere^(6)^.

In the USA, to be approved as a health claim by the USA Food and Drug Administration (FDA), an SSA (significant scientific agreement) claim must be supported by the totality of publicly available scientific evidence for a substance–disease relationship^(43)^. In this regard, for the highest level claims such as SSA claims, the FDA, EFSA and UKNHCC systems are similar^(44)^. However, a clear difference from the scope of claims in Europe (EU and UK) is that an SSA claim can also refer to disease reduction, whereas Article 14(1)(a) claims are limited to disease risk factor reduction (e.g. a reduction in blood cholesterol concentration rather than coronary heart disease per se).

An example of an authorised SSA health claim from FDA is as follows:

‘Adequate calcium and vitamin D as part of a healthful diet, along with physical activity, may reduce the risk of osteoporosis in later life’^(45)^.

In contrast, the EU/UK claim is stated in two distinct sentences. The link to the disease must come from separate evidence relating the risk factor to the disease.

‘Calcium and vitamin D help to reduce the loss of bone mineral in post-menopausal women. Low bone mineral density is a risk factor for osteoporotic bone fractures’^(31)^.

The FDA also allows *qualified* health claims^(46)^. A ranking system for the strength of the evidence for qualified claims was established by the FDA, with strength ranging from moderate/good, low, to lowest. To help ensure that these qualified claims are not misleading, they must be accompanied by qualifying language to accurately communicate to consumers the lower level of scientific evidence^(43)^.

An example of an FDA qualified health claim is:

‘Scientific evidence suggests, *but does not prove*, that whole grains (three servings or 48 g/d), as part of a low saturated fat, low cholesterol diet, may reduce the risk of diabetes mellitus type 2’^(47)^.

A further type of USA health claim (named *FDAMA claims* after the FDA Modernisation Act) can also be authorised based on an authoritative statement from an appropriate scientific body of the USA Government or the National Academy of Sciences or any of its subdivisions^(48)^. FDAMA claims, which can only be used on conventional foods and not food supplements, require foods carrying health claims not to exceed the disqualifying amounts of nutrients that may increase the risk of a disease or health-related condition in the general population.

One example of an FDAMA claim is:

‘Diets rich in whole grain food and other plant food and low in total fat, saturated fat, and cholesterol may reduce the risk of heart disease and some cancers’

The *Japanese* system has two major categories of claims^(49)^. The first category, ‘Food with Nutrient Function Claims,’ is a claim which is similar to the Article 13(1) function claims in Europe (see ‘The nature of the evidence base underpinning health claims in the EU and UK’ section). Two separate statements must be made (the function claim and the caution) as in the following example for folic acid:

‘Folic acid is a nutrient that helps red cell formation. Consuming a large quantity of this product does not cure disease or promote health. Please use no more than the recommended daily intake’.

The second category of Japanese claims is similar to the Article 14(1) claims in Europe and is known as ‘Food for Specified Health Uses’ (FOSHU). FOSHU foods (first introduced in 1990) are those that contain dietary ingredients that maintain and promote health, and improve health-related conditions. Generally, these claims are not allowed to state that foods can be used to treat or alleviate symptoms of a disease^(4,49)^.

There are two main types of FOSHU claims: (1) ‘*Regular FOSHU*’ and (2) ‘*Reduction of disease risk FOSHU*’.

*Regular FOSHU* claims refer to claims which have sufficient scientific evidence as well as an established mechanism. There are two sub-divisions: ‘*Standardized*’ FOSHU, are claims with sufficient scientific evidence and for which a licence or approval is granted and ‘*Qualified* FOSHU’ claims are those where the level of supporting evidence is considered insufficient or where there is adequate evidence but no established mechanism. Qualified FOSHU should include the following statement: ‘grounds for this effectiveness have not necessarily been established’. These categories were created in order to incentivise applicants and, although the scientific evidence is assessed in the same way, they are judged differently based on lesser strength of the evidence.

Only two *Reduction of disease risk FOSHU* claims are, so far, authorised in Japan^(49,4)^. A translation of one example is shown:

‘This product contains adequate calcium. Intake of a proper amount of calcium contained in healthy meals with appropriate exercise may support healthy bones of young women and reduce the risk of osteoporosis when aged. Diseases are generally caused by various factors. Excessive ingestion of calcium will not eliminate the risk of developing osteoporosis. Daily intake of calcium from the FOSHU products should be between 300 and 700 mg’.

Risk assessment in Japan is separated from risk management and, as in EU/UK, the wording of the claim must also be approved by the risk managers^(4,49)^. A number of conditions must be met: the FOSHU product must be evaluated using efficacy studies; demonstrate absence of any safety or toxicity issue; provide analytically assured levels of the functional component and must have an appropriate nutritional profile. The latter is of interest in comparison with EU/UK claims, where the possibility of introducing nutrient profiling for foods displaying health claims is included within the existing Regulation, but has yet to be enacted and is still under consideration (see ‘The strengths and challenges of the health claims process followed in the EU and UK’ section). Delays in reaching agreement within the EU on a nutritional profiling approach for products carrying a health claim have received critical comment^(5)^

Australia and New Zealand share common food standards managed through the authority of Food Standards Australia New Zealand (FSANZ)^(50,51,52)^. Their systems were reviewed recently with the aim to produce a comprehensive framework that protects and assists consumers, yet provides opportunity for industry to submit applications without incurring the lengthy time and cost implications of producing a full dossier. FSANZ allows two types of claims, known as *high* and *general level claims* ^(53)^.

*High level health claims* must be based on food–health relationships pre-approved by FSANZ. They refer to a nutrient or substance in a food and its relationship to a serious disease, or risk factor for disease. Examples include: *Diets high in calcium may reduce the risk of osteoporosis in people 65 years and over, and Phytosterols may reduce blood cholesterol*. *General level health claims* refer to a nutrient or substance in a food, or the food itself, and its effect on health but not on any serious disease. An example includes such as: ‘*calcium for healthy bones and teeth’* ^(54)^.

Since 2016, food businesses wanting to make a *general level health* claim can base their claim on one of the 200+ pre-approved food–health relationships^(55)^. Alternatively, in a process which differs from the other Frameworks covered here, *food companies can self-substantiate* a food–health relationship by following a prescribed process for systematic review of relevant evidence, the outcome of which they can be asked to produce at any time^(56)^. The process for conducting the review is set out by FSANZ and guided by similar principles to those of the EU/UK efficacy trials. FSANZ maintains a public record of self-substantiated general level health claims, but does not further investigate the merits of food–health relationships notified this way^(53)^. Surveys in Australia and New Zealand have shown self-substantiated claims are based on less robust evidence than health claims which have pre-approval^(56)^. Examples taken from the Register (https://www.foodstandards.gov.au/industry/labelling/fhr/Pages/default.aspx) are as follows:

Hydrolysed collagen reduces fine lines and wrinkles

Increasing grain fibre intake reduces feelings of hunger

A further difference from the UK and EU approaches, but similar to Japan, is that all foods carrying any type of FSANZ health claim must meet certain compositional requirements, including a nutrient profiling system based on that used in the UK to regulate advertising to children^(57,58)^.

## Conclusions on the different types of health claims across the world

The cross-country comparison shows that EU and UK only authorise claims with strong scientific agreement, whereas other countries have systems for authorising claims ranging from the strongest scientific agreement to lesser levels where the evidence is not so strong (USA, Australia, New Zealand and Japan). Australia and New Zealand also allow self-substantiation of claims in some cases. Qualified and self-substantiated claims appear to offer greater incentivisation for the food industry to produce more products with qualified claims, as reflected in the number of such claims recorded for FSANZ^(52)^.

However, the consumer studies summarised in ‘The strengths and challenges of the health claims process followed in the EU and UK’ section show that the systems operating in some countries with more types of claims tend to confuse consumers rather than help them. More evidence is required to establish whether or not qualified claims are beneficial for consumers. If proved beneficial, the challenge remains to express the claim in a way that clearly reflects the level of supporting scientific evidence and to set appropriate scientific criteria for the assessment of evidence that is considered sufficiently robust to justify a qualified claim.

## The strengths and challenges of the health claims process followed in the EU and UK

### Strength: thoroughness and transparency of the authorisation process based on the totality of the evidence base

It is fundamental that scientific risk assessment of the totality of the evidence base is conducted by independent scientists and that it is open and transparent^(14,59)^.

### Strength: separating risk assessment from risk management

To avoid the risk of bias, the risk assessment process has been clearly separated from risk management. To this end, EFSA’s remit with respect to the assessment of health claims for foods is based upon the EFSA Founding Regulations^(60)^. This made it clear that EFSA does not authorise nutrition and health claims, set labelling requirements, make recommendations to consumers or monitor and assess consumers’ behaviour or societal and economic aspects. Therefore, in the system adopted in the EU, EFSA is responsible for all aspects of risk assessment and the EC, and the Parliament and Member States of the EU are responsible for risk management. In the UK, a similar process is now followed whereby the UKNHCC carries out the risk assessment and the Four Nations group (under the auspices of the devolved Departments of Health) must then consider the consequences of allowing the health claim to be made in the context of the whole diet, i.e. what effect this might have on the populations consuming the food or constituent^(61)^.

Two examples of how the multi-tier EFSA system has worked are given below:

#### Glucose and energy

A set of claims, mostly related to the contribution of glucose to energy-yielding metabolism, were assessed by the EFSA NDA Panel under Article 13(5) and given positive opinions as claims based on newly developed scientific evidence^(62)^. However, in 2015, the EC took the decision not to authorise these health claims because they were considered to be ambiguous and misleading as they conflict with public health advice to reduce sugar consumption^(63)^.

#### Caffeine and alertness

The Article 13(5) health claim for the effect of caffeine on ‘alertness’ was originally given a positive opinion by EFSA and a draft regulation proposing authorisation was then drawn up by the EC^(64)^. However, the draft regulation was subsequently rejected by the European Parliament (EP) in Strasbourg under the scrutiny procedure, presumably because the EP did not consider it wise to encourage caffeine-containing drinks with high energy and sugar content to be consumed by all sectors of the population, especially adolescents. The health claim has, therefore, not been authorised by the EC.

## Strength: provision of extensive guidance on submissions for health claims

There is no doubt that in the early years of the EU Nutrition and Health Claims Regulation, stakeholder (normally the Food and Drink Industry) perception of the process was not always very positive. However, since 2006 considerable work has been done with industry and other stakeholders to clarify some of the complexities of the process and improve clarity of the associated guidance^(14,59)^.

A notable development has been the extensive published guidance from EFSA (very recently updated), on the preparation and presentation of a health claim application to help encourage high quality submissions and also provide more transparency about the assessment process^(13)^.

As well as general guidance, EFSA has also published several specific guidance documents based on its experience with applications received over a number of years^(65)^. These focus on specific physiological systems and their measurement, in order to help guide the selection and use of appropriate risk markers in human efficacy trials.

Notwithstanding the advances that have been made in guidance on health claim submissions, considerable challenges for those submitting claims continue to be reported and debated. This is confirmed by the relatively low success rates for submissions, e.g. 34 % for risk-reduction claims (see Table 1 on page 00).

## Challenge: conducting efficacy trials of foods

The conduct of successful food and diet trials is more demanding to undertake than drug trials so particular consideration needs to be given to aspects of study design that can help reduce poor compliance and high drop-out or failure of the trial. Some of the particular challenges for diet *v*. drug trials and their implications are summarised in Table 3.

**Table 3.** Differences between drug and diet or food trials in human subjects

Challenges in diet trials *v*. drug trials	Implications
Smaller effect size for diet	Requires larger sample size to demonstrate significant effect
Selecting suitable placebo food or diet for control group	Control diet or food needs to have similar energy and nutrient compositions as those for the test, but without the active constituent. Composition is not generally a problem faced with drug trials.
Consideration for conditions of use	Requires standardisation of background diet which may adversely affect adherence and compliance
Outcome measures for food and health claims based on reduction in a risk biomarker; not a change in a disease state (as is case for drugs).	Risk markers are not always available for every health effect that may be mediated by food, or the effect may be in an inaccessible location (e.g. liver or gut, rather than blood or urine)
Variation in risk marker measurements due to impact of metabolic or nutritional status at time of measurement (e.g. glycaemic index, blood lipids).	Standardisation of fasting or feeding and exercise may be required. Repeat measures of outcome markers may be needed throughout the study to achieve reliable results.
Defining and recruiting a healthy population	Over 60 % of many European populations have abnormal values for many of the clinical criteria used in recruiting to food studies (blood cholesterol, BP, fasting glucose). This limits the recruitment process and applicability of the findings as the Regulation specifies relevance to a ‘normal healthy population’.
Recruitment bias due to nature of the healthy profile of volunteers	Volunteers may have particular interest in diet and health, and consequently healthier lifestyles, resulting in risk marker values unrepresentative of the range observed in a ‘normal healthy population’.

Our view reflects that of de Boer^(5)^ in that the demanding nature of efficacy trials for foods compared with that for drugs result in higher costs, large drop-out rates and loss of study power, with greater risk of null findings.

With increasing emphasis on more holistic concepts of healthy diets^(5,35)^ and dietary profiling, there is a risk of declining interest in investigating the potential for single foods, food components and supplements to have measurable impacts on health markers. There may be a need for a shift in conceptual thinking about the impact of individual foods on health and wellness, which may not fit the original ‘functional food’ approach which underpins present-day approaches to health claims assessment. De Boer^(5)^ considers a resilience model^(66)^ that might better reflect the subtle, widespread and (often) transient physiological effects of food on health which cannot be adequately measured during many short-term efficacy trials, but which nevertheless can have an impact on long-term health.

In the meantime, many of the existing challenges outlined in Table 3 remain to be resolved, with some of these discussed in further detail below.

## Challenge: developing clearly defined risk biomarkers that can be used in food efficacy studies

The primary role of EU and UK health claims legislation is to protect individuals by allowing claims to be made only where there is robust evidence of a positive effect of a food or its constituent(s) on the health of the target population group under the proposed conditions of use. Function claims based on essentiality of a nutrient are relatively straight-forward to justify, but collation and assessment of evidence about function claims for non-nutrients and reduction of disease risk claims can be more complicated. The latter are of growing importance, given current and predicted demographics and the important goal of maximising healthy ageing. Such claims can only be made for changes in risk biomarkers, of which very few are sufficiently validated to date.

It is clear that, to advance the scientific evaluation of risk reduction health claims, there is need to develop markers that characterise the full spectrum of health in a population, including biomarkers that reflect the ageing process. These need to go beyond individual markers, e.g. LDL-cholesterol, towards algorithmic predictors of disease risk using multiple markers of metabolic health. This could also include validation for the use of candidate biomarkers of ageing, including biochemical measures (e.g. IL-6, CRP and HbA1c), molecular markers^(67)^ or epigenetic biomarkers^(68)^. Such developments could eventually contribute to an advancement in providing dietary advice and specific foods to support health, based on more individualised assessment, known as personalised or precision nutrition This refers to the use of a range of factors, including dietary habits, genetics, eating patterns, circadian rhythms, health status, socio-economic and psychosocial characteristics, food environments, physical activity and the gut microbiome to characterise an individual’s metabolic phenotype and genotype^(69,70,71,72)^ (van den Broek, 2017 #185, NIH Nutrition Research Task Force, 2019 #42).

Clearly, health claims, as they currently exist, have limited utility in the spectrum of personalised and precision nutrition. Their primary use is to provide consumers with additional information about the benefits of certain foods, as well as the opportunity to distinguish between different brands of a similar food group and to select the ones that are more likely to promote health. However, in the future, there may be an opportunity for niche markets whereby specialised products are available for groups of individuals, who are currently healthy, but have been identified as having higher nutrient requirements (e.g. folate in relation to an MTHFR mutation

j) or are at a higher risk of certain diseases such as cardiovascular disease (e.g. high saturated fat intake and risk of elevated LDL-cholesterol).

## Challenge: defining a healthy population and future scenarios

The focus of the UK and EU legislation regulating health claims (as currently formulated) is the maintenance of health via reduction of disease risk, rather than disease reduction *per se*. Hence, the rationale in the regulation which determines that subjects recruited into efficacy trials should be from the normal healthy population. However, this is challenging for many reasons because the definition and measurement of ‘healthy’ is open to interpretation. As people age, there is a steady transition from ‘normal healthy’ towards the disease end of the healthy-to-disease spectrum. This process is very variable depending upon environmental factors such as body weight and life-long habits such as inactive lifestyles, smoking and stress. However, those whose health measures are moving towards the unhealthy range are precisely the population who may wish to alter their diets and lifestyles and be open to the use of foods with authorised health claims. This particularly applies to older, health-conscious individuals, a demographic which is on the increase across most developed countries.

Selecting subjects for efficacy trials of foods or constituents to support a particular health claim will also be challenging if the recruited individuals need to be within the ‘healthy weight’ range. There is a shrinking population that can be classified as healthy using strict criteria of healthy body mass index, blood pressure, blood lipids, no health problems, no medication and being physically active. In England, over 60 % of adults are overweight or obese^(73)^ and approximately 26 % are hypertensive^(74)^, while 40–45 % have raised cholesterol^(75)^. As a consequence, the generalisability of the findings from ‘healthy’ subjects to populations with more heterogeneous health profiles may be very limited. Further, recruitment of only ‘at-risk’ individuals confines the relevance of the findings to this group and would be of limited validity to others whose aim is to prevent reaching an at-risk category.

The Academy considers that the definition of ‘normal healthy’ needs to be more nuanced to ensure that the study group reflects the likely target population for the food product. However, limited progress has been made in developing suitable pre-disease markers that can replace the present status quo. There is need for better research to identify population groups at different states of the health–disease continuum^(76)^ who might benefit (more) from particular foods or drinks. If this were achieved, products bearing authorised health claims for specific population groups could be beneficial for people in the pre-disease state who potentially have the most to gain from consuming the foods.

## Challenge: ensuring the composition of a food bearing a health claim is consistent with generally accepted nutrition principles

The issue of nutrient profiling is also a topic which has been much discussed in relation to health claims^(5)^ It has been defined as the science of classifying or ranking foods according to their nutritional composition for reasons related to preventing disease and/or promoting health^(77)^.

According to Article 4 of the 2006 EU Regulation^(2)^, ‘*a nutrition or health claim should not be made if it is inconsistent with generally accepted nutrition and health principles or if it encourages or condones excessive consumption of any food or disparages good dietary practice’*. Acceptance of these principles has been illustrated by the European Parliament’s decisions to disallow claims related to health effects of glucose and caffeine on alertness, despite the scientific assessors having approved the claims from a risk assessment perspective. The 2006 Regulation specified that a nutrient profile should be in place (by 2009) to determine conditions under which a nutrient or health claim can be made on a food or drink. But this has yet to happen.

An evaluation of the 2006 EU Regulation, carried out in 2020 by the European Commission’s Fitness Check^(23)^, highlighted that the objective of Article 4 has been hampered across the EU by the failure to relate the use of health or nutrition claims to the nutrient composition (profile) of foods and drinks. An EFSA consultation on nutrient profiling for several purposes, including claims, was launched in November 2021 and closed in January 2022^(78)^. The outcome has not yet been reported.

A number of other countries (e.g. Australia, New Zealand and Japan) have developed formal schemes which involve nutrient profiling of foods as a determinant of whether they can carry authorised health claims (see ‘The strengths and challenges of the health claims process followed in the EU and UK’ section).

There is, however, uncertainty about the impact of introducing a formal nutrient profiling scheme for nutrition and health claims in the EU and/or UK on the quality of consumers’ diets. Studies to date indicate that the impact will depend upon which scheme is used for profiling. The challenge may in part relate to the greater diversity of diets across the EU and/or inability to reach sufficient consensus on the need for profiling.

The main challenge is to reduce the potential of a food legitimately carrying a health claim to adversely affect overall dietary balance as a result of other aspects of its composition (e.g. the food might be a rich source of a micronutrient with an authorised health claim but have a very high content of, say, saturated fat and/or salt).

## How do consumers view and use health claims?

### Evidence from scientific studies

The regulation of health claims was always intended, primarily, to regulate the market and protect consumers from unscrupulous claims. So how do consumers view and use health claims? Various studies in Europe and beyond have examined the understanding and acceptance of, as well as actual use of, nutrition and health claims by consumers. Whilst the EU and UK legislation makes a clear-cut distinction between nutrition claims, function-related health claims and disease risk reduction claims, an early review concluded that consumers probably do not make the same distinction^(79)^.

There is, however, evidence that consumers pay attention to nutrition and health claims and that their presence can sometimes initiate behaviour change (e.g. encourage people to eat ‘low fat’ products more frequently). For example, a meta-analysis of seventeen controlled experiments, conducted in artificial settings, concluded that nutrition and health claims have a substantial effect on purchasing and/or consumption of specific products and therefore dietary choices^(80)^. However, impact on purchasing appears to be higher amongst those who already tend to buy healthier foods and have an interest in nutrition^(81)^ or those with higher health motivation^(4)^.

The use of health claims is influenced by a variety of factors. These include personal knowledge and familiarity with the information or brand, relevance to the individual of the described benefit, characteristics of the product and the way the claim is presented (e.g. wording and visual aids such as symbols)^(82,83,84)^. Cultural differences also clearly exist across Europe^(85,86,87,88)^. For example, a large survey of consumers conducted as part of the *CLYMBOL* project (funded by the EC) within ten European countries found consumers in Spain and Greece used health claims most during purchasing, while consumers in the Netherlands used them least^(83)^. Of course, most research to date has been conducted in artificial settings. Whilst there have been some studies conducted in more natural settings these have reported smaller effects. Further research is therefore needed to assess impact on real world dietary choices^(80)^.

Reference was made in ‘Types of health claims in other countries – how do they differ from EU and UK’ section to the different types of health claims used outside Europe. In the USA, research has suggested that people do not perceive significant differences between the three levels of the USA qualified claims and fully authorised (SSA) health claims^(89)^. An FDA investigation of consumer perception of health claims concluded that none of the tested communication schemes was consistently capable of highlighting the distinction between SSA claims (equivalent to EU claims) and claims with the next (lower) level of scientific evidence^(90)^.

## Challenge: making the wording of health claims more understandable for consumers

Consumer understanding is a key aspect of the EU (and now UK) Regulation^(2)^, which states that ‘*use of nutrition and health claims shall only be permitted if the average consumer can be expected to understand the beneficial effects as expressed in the claim’*. The Regulation requires the use of the official (authorised) wording for each claim when it is used on products or in other communications, such as advertising and marketing, although there is some flexibility. Food companies are permitted to make some relatively minor changes to aid consumer understanding, taking into account factors such as linguistic and cultural variations and the target population, as long as the original meaning is retained and the modified version does not mislead^(91)^. Differences in culture and language among different member states can, however, present challenges, and wording of claims and their interpretation by consumers has been raised as a major concern^(92)^. The formulation of wording of health claims is also perceived to be one of the biggest challenges faced by the food industry in complying with the Regulation^(93)^.

The core of the problem has been that the specified wording that emerges from the regulatory processes is sometimes considered overly technical, lengthy and/or uses words such as ‘normal’ which do not motivate consumers. This does not align well with the type of plain English wording that industry would prefer to use as a communication tool and that consumers would like to see on foods. The current list of authorised function claims consists of statements that range from simple to more technical use of language (see ‘Regulatory frameworks and types of health claims’ section for examples). Research suggests that shorter and simpler claims are better able to promote consumer understanding and more likely to encourage engagement with product packaging whilst shopping^(84)^.

Although the scientific rigour of the approval process in the EU and UK should provide consumers with assurance about the validity of authorised health claims, some consumers view health claims with scepticism. Findings from *Health Claims Unpacked* showed a high proportion of consumers surveyed in the UK, France, Germany and Poland perceive health claims simply as a marketing tool^(94)^. This project, funded by EIT-Food, gathered data from large numbers of consumers in these four countries, and found a distinct lack of preference for authorised claim wording compared with alternative, similar wording^(94)^. For example, when asked to choose their own wording for a claim from a selection of relevant words and phrases, the word ‘normal’ (which features in many authorised claims written in English) was only chosen by a very small proportion of participants, with most opting for alternatives such as ‘healthy’. Health claims that refer to compounds or substances (as well as health benefits) which are familiar to consumers (e.g. Ca and its effect on bone health) were considered trustworthy, leading consumers to believe more in the healthiness of a product and increase purchasing intention^(4,95)^.

The CLYMBOL Project^(4,95)^ also concluded that communication of health claims should be kept simple, clear and scientifically sound, yet phrased without using overly complex scientific language or regulatory jargon, in order to be meaningful^(83)^.

Seventeen EU Member States agreed an informal set of general principles linked to the wording of claims in 2012^(96)^ and, in some countries, further advice has since been provided (e.g. UK^(97)^, Belgium, Italy and The Netherlands^(98)^). However, a desire for clearer guidance for industry and other stakeholders around acceptable rewording of claims has recently been identified^(94)^. The UK’s exit from the EU means that the UK government and devolved administrations could re-consider the approach taken for the wording of health claims specific to the UK, with a view to assisting consumer understanding (while at the same time not enabling the sale of products whose messages mislead). Further research is needed on the impact of the wording of health claims on consumer understanding and decision taking (and specifically whether reworded claims are perceived to have a different meaning or result in healthier choices) among different groups.

## Challenge: understanding the implications of the ‘health halo effect’ and other misperceptions around products bearing health claims

Although the specific conditions of use for some authorised claims may aid in ensuring that clearly unhealthy products cannot bear such claims, the absence of a formalised nutrient profiling approach associated with the legislation may make it more difficult for consumers to decide whether products with specific ingredients that may offer a health benefit are a good addition to their diet overall^(99)^.

As described above, several studies have shown consumers to be more positive towards a product when a nutrition or health claim is present, although this appears stronger for disease risk reduction claims and claims that are shorter in length^(100)^. However, some consumers may fall prey to cognitive biases associated with nutrition and health claims. Whilst some consumers appear to understand that rigorous scientific assessment is required before a claim can be authorised, many see health claims as giving a ‘halo’^(101,102)^ or ‘magic bullet’ effect to a product, which can distort their perception of the healthiness of the food^(82)^. ‘Positivity bias’^(103)^ is a term used to describe these types of misperceptions which appear to reinforce the case for foods carrying health claims to be subject to nutrient profiling thresholds. In contrast, concern has also been expressed that health claims may discourage selection of healthier foods by some due to a perceived negative link with taste (i.e. healthier foods are less tasty of unhealthy foods are more palatable). This was supported by a recent study in Denmark showing products defined as unhealthy and health neutral were chosen less frequently if they carried reduction claims (e.g. ‘Reduced salt content’, ‘Contains less sugar’ and ‘Fat free’) compared with non-claiming products^(104)^.

## Health professionals and health claims

### Why do health professionals need to know about claims?

The present paper has outlined the importance of obtaining strong, consistent evidence for effects of foods or food constituents on health and on risk markers for disease, and the rigorous processes that are used for assessing the strength and quality of evidence needed to make a health claim, and the challenges involved (see ‘The strengths and challenges of the health claims process followed in the EU and UK’ section). The paper has also illustrated the challenges experienced by consumers seeking to use health claims to identify diets and foods which may improve their health, including foods that can reduce risk factors for chronic illnesses, such as heart disease, osteoporosis or diabetes. As has already been described in ‘How do consumers view and use health claims?’ section, many consumers report finding the wording of health claims unclear or lacking sufficient motivational content. Others perceive health claims with scepticism, whereas some are positively influenced by the presence of a health claim on a product such that they mistakenly assume the product has nutritional benefits, in addition to the claimed effect, that are not warranted.

These days, in the formal training received by Registered Dietitians and Registered Nutritionists, there is typically some inclusion of pertinent food legislation, such as the Nutrition and Health Claims Regulation and the associated processes discussed here, but the extent is likely to vary between courses. Other types of health professionals and those working on the periphery of the food and health sector are less likely to be familiar with the rigorous nature of the approval process and the complexity of the Regulation and associated Guidance.

In professional practice, dietitians and nutritionists may work in a number of settings where knowledge of the Nutrition and Health Claims Regulation and Guidance is of particular importance, including

- academic or clinical trial researchers conducting efficacy trials on behalf of industry sponsors seeking independent verification of their product efficacy as part of a Health Claim.
- those working with food and drink businesses to help them to submit a health claim application and/or responsibly use rigorously authorised health claims in marketing or advertising so that consumers can trust the messages.
- those working with patients or the general public to help them identify and understand the potential benefits of foods that carry authorised health claims.

There is considerable potential for more and better communication about the EU and UK Regulations including the rigour of the claim approval process itself, which could help reduce misunderstanding and lack of trust. Health professionals (especially dietitians and nutritionists with formal training in the topic) could play a key role in this regard, educating people about the existence and purpose of the Regulation and the rigour of the approval process and encouraging them to use authorised claims on food labels to support healthier dietary choices.

The efforts of nutritionists and dietitians working directly with the food industry is key to developing trust in health claims. Some are food industry employees (mainly in larger businesses), while others act as consultants providing specialist advice and guidance, or run their own food businesses. As well as supporting submission of new health claim applications, they typically have an important role in ensuring food and drink companies make health-related statements about their products which are authorised claims and comply with the conditions of use. This includes ensuring that marketing messages are aligned with the authorised wording of the relevant health claim or that the food contains the required amount of nutrient or active ingredient, thereby helping to develop greater trust over time and protecting consumers.

The Academy considers there has been insufficient attention paid to the complexity and implications of the EU and UK Nutrition and Health Claims Regulations for professional practice in nutrition and dietetics and considers that nutrition, dietetic and potentially other health professionals, such as doctors and nurses, should ensure they are up to date in their knowledge concerning use of health claims. These professionals are in a prime position to provide clear advice to patients, other health professionals in practice and in training and to other non-professionals seeking advice and guidance on the workings and implications of this Regulation.

As previously mentioned, all nutrition or dietetic courses accredited by the relevant regulatory and professional bodies (BDA, AfN) should ensure that their criteria for accreditation of their relevant training programmes includes an appropriate understanding of the pertinent legislative context. Approved continuous professional development provision should be available for practitioners working in this area (including doctors and other health professionals giving dietary advice) to attain and maintain their knowledge and understanding of the Nutrition and Health Claims Regulation and implications for their professional practice

The Academy is aware that some institutions may lack sufficiently qualified individuals able to deliver this specialist material and propose that a programme of training delivered by relevant individuals within the ANS member bodies should be developed to bridge this gap and support continued professional development.

### Article 12(c) in the nutrition and Health Claims Regulation

If professionals working in nutrition and health want to become more actively engaged in communicating how health claims can contribute to the process of making healthy food choices, they need to be aware of a specific Article (12(c)) in the Regulation that regulates what they can say and do in the context of commercial communications (marketing) on foods carrying a health claim. The context for this Article can be found in the Regulation’s Article 1, which states that the legislation covers all claims on food products in commercial communications.

Article 12(c) ‘prohibits health claims that make reference to recommendations of individual doctors or health professionals (or to an association other than a national association of medical, nutrition or dietetic professionals or a health-related charity)’.

The GB Guidance^(11)^ says in Section 4.5 *‘Our understanding is that this prohibition was put in place due to concern that, in commercial communications, the added weight of perceived professional expertise might unduly influence consumers, and the objective of the Regulation is that consumers should not be misled in any way.’*

The GB Guidance^(11)^ also explains the implications of Article 12(c) for medical, dietetic and nutrition professionals in more detail. It makes the clear distinction between commercial and non-commercial communications. Article 12 (c) applies to all commercial communications, examples of which include ‘*any form of product labelling or packaging to be delivered as such to the final consumer, product specific advertising in any form, including in print, broadcast, internet or direct mail, promotional features in print media, in-store promotions and food business social media*’. It does not apply to non-commercial communications. These include dietary guidelines or advice issued by public health authorities and bodies, such as that to eat at least five portions of fruits and vegetables a day. Also not within scope of the regulation are advice given by nutrition professionals to patients or clients during consultations, information in scientific publications textbooks and lectures, or ‘business to business’ content such as press releases, brochures or websites where the final recipient is a business, healthcare professional or journalist rather than the general public. The GB Guidance gives examples of how authorised claims should be communicated in commercial settings.

The Academy recognises that a growing number of nutritionists and dietitians work in commercial settings and wish to refer to authorised health claims to inspire confidence in the processes outlined in this position paper. However, they are confused about the apparent prohibition of Article 12 (c). A small survey has been undertaken of professionals working in the sector to explore this topic. The preliminary unpublished findings reveal widely differing interpretations of the current GB Guidance^(105)^. These results confirm that further clarification is needed on what nutrition and dietetic professionals working in commercial communications settings are reasonably permitted to do. In developing future guidance, there is a need to ensure proportionate interpretation of the regulations to champion the important role of qualified nutrition/dietetic professionals in consumer communication, and encourage the food industry to continue engaging with the nutrition profession.

A further issue that arises in the context of commercial communications is the wording in Section 10 of the GB Guidance^(11)^ which states that :

Celebrity endorsements do not appear to fall within the scope of the prohibition in Article 12(c) (unless the celebrity is a doctor or health professional).

Therefore, the Academy is concerned that the current situation appears, by default, to allow non-professionals (such as ‘influencers’ and ‘celebrities’) with limited or no professional training in nutrition science to provide high profile endorsements of authorised health claims within advertisements and marketing materials for foods and food supplements. At the very least, celebrity endorsement may give undue weight to an authorised health claim and the influence such individuals can have on the public is often far greater than any individual health professional. Even more serious, lack of training in nutrition science and the associated legislation may risk influencer and celebrity endorsement being associated with non-authorised and potentially unsafe or misleading claims.

While it is clear that the legislation on health claims came into force prior to the full evolution of social media and associated marketing of products by influencers, it is also the case that diets, foods and dietary supplements have now become a major segment for income generation via social media. This presents the potential to undermine the main principles of the Nutrition and Health Claims Regulation, which is precisely to help/protect the consumer by distinguishing fully evidenced authorised claims from those which have limited or no scientific validity. It also creates a non-level playing field where qualified nutrition/dietetic professionals, whose practice is governed by Codes of Practice and professional registration, are not permitted to communicate authorised health claims to consumers in commercial communications, while unqualified individuals can. This situation appears to be inconsistent with the aims and objectives of the Regulation and potentially undermines the principles of evidence-based and proportionate regulation. The Academy considers that the GB Guidance associated with Article 12(c) needs to be reviewed to help ensure consumer protection and consistent interpretation.

## Summary with strengths and challenges

This position paper from the Academy of Nutrition Sciences is the second in a series of papers which describe the nature of the scientific evidence and the processes that underpin nutrition recommendations for health. This paper summarises the scientific assessment process for health claims used by the EFSA, which has been adopted as the basis of the process now being used by the UK Nutrition and Health Claims Committee (UKNHCC) for the assessment process in Great Britain. Consideration is given, as appropriate, to other countries’ systems, namely USA, Japan, Australia and New Zealand. The paper raises some of the challenges of performing human efficacy trials for foods and food constituents (as opposed to drugs), which form the main evidence base for EFSA and UK Nutrition and Health Claims decisions. Aspects of consumer understanding and perception related to health claims are also considered, as well as the implications of the Nutrition and Health Claims Regulation for health professionals working with the public and the food industry.

The Academy notes that the approval of a proposed health claim in the EU or UK requires a submission portfolio (dossier) which includes evidence from human efficacy trials in healthy subjects. These trials need to demonstrate that consuming the food or constituent, which is the subject of the claim, has measurable beneficial effects on a defined physiological function or on a surrogate marker of disease (i.e. the health effect being claimed). Other types of evidence such as mechanistic studies can be used to support the efficacy trial data.

The Academy recognises, as *strengths*, the thoroughness and transparency of the EFSA assessment approach, now in use in the UK, and the fact that it is kept separate from the risk management process. Another *strength* is the extensive guidance which has been developed by EFSA over the past 15 years to encourage high-quality submissions for health claims and to ensure transparency. Other countries, e.g. USA, Australia and New Zealand, in addition to fully authorised claims, have introduced several other categories of health claims (e.g. qualified claims) which require less rigorous substantiation than their authorised claims, coupled with a form of wording that communicates the nature of the assessment process. This alternative approach appears to make claims easier to achieve and encourages industry innovation. However, is it not clear whether or not these less rigorous processes offer potential risk to the public through use of products whose constituents have not always been fully assessed for their efficacy and by the public’s misunderstanding of the strength of the claim.

The Academy recognises four main *challenges* in undertaking efficacy trials to support new health claim applications:

1. The added complexity of undertaking efficacy trials for foods in human subjects compared with protocols for drug trials.
2. Defining a ‘healthy population’ for the purpose of these trials.
3. Developing clearly defined biomarkers for some outcomes to support robust efficacy trials.
4. Ensuring the nutrient composition of a food bearing a health claim is consistent with ‘generally accepted nutrition principles’.

Although the Nutrition and Health Claims Regulation is primarily directed at protecting the consumer from harm, the Academy has considered findings from EU consumer research that indicate some *challenges* that need to be overcome to help consumer understanding:

1. Making the wording of health claims more understandable for consumers.
2. Understanding the implications of the ‘health halo’ effect around food and other misperceptions around products bearing claims.

The Academy recognises that much of the consumer research concerning different categories of health claims used globally, as well as the optimal wording to communicate these claims, is still at a relatively early stage, with findings from the latter type of research reflecting the wide cultural and socio-economic variation which exists across Europe and globally.

In relation to health professionals, the Academy recognises two main *challenges*. It considers that Registered Nutritionists and Registered Dietitians (and other health professionals giving dietary advice) should be aware of the rigorous, transparent assessment of the evidence behind authorised health claims (within the confines of the Regulation), as well as the implications of the Regulation for their professional practice and ensure that they have a level of understanding that is appropriate to their professional roles. The Academy notes that Article 12(c) of the Nutrition and Health Claims Regulation does ‘not allow claims which make reference to recommendations of individual doctors or health professionals in commercial communications…’. The Academy recognises that the interpretation of this Article remains controversial and confusing for those nutritionists and dietitians directly affected by it (e.g. those who work with or for the food industry and contribute to the communication of authorised health claims).

Counterintuitively, no such prohibition applies to endorsements by non-experts in nutrition and health, such as ‘celebrities’ and ‘social media influencers’. This is a *challenge* to the communication of evidence-based information, so needs review to help ensure consumer protection and consistent interpretation.

## Recommendations

The Academy makes the following *recommendations* which relate to the Nutrition and Health Claims Regulation:

1. The Academy considers that a clear consensus concerning the scientific classification of ‘healthy’ and ‘unhealthy’ individuals in efficacy trials has not yet been fully explored or agreed. Development of clearly defined biomarkers that can support such definitions and which may also be used as outcome biomarkers in such trials for health claims are required. The Academy *recommends* that scientific societies, organisations and funding agencies consider these as priority topics for scientific meetings and for future research programmes.
2. The Academy considers that the implications of different health claim category options (e.g. qualified claims) available in some parts of the world, as well as consumer understanding of the required wording of health claims, need to be better understood and *recommends* that further behavioural research is required: (i) to determine the relative benefits, to the consumer, of having health claims based on differing levels of rigour and certainty, and (ii) to promote the use of wording for claims that supports better understanding of the claim by consumers.
3. To support relevant training of Registered Nutritionists and Dietitians, the Academy *recommends* that the relevant regulatory and professional bodies (AfN; BDA) ensure that their criteria for accreditation of their relevant training programmes include an appropriate understanding of the pertinent legislative context and that approved continuous professional development provision is available for practitioners working in this area (including doctors and other health professionals giving dietary advice) to attain and maintain their knowledge and understanding of the Nutrition and Health Claims Regulation and implications for their professional practice. By introducing this into their practice, they would help to inspire trust in the health claims process.
4. To support clearer and consistent interpretation of the Guidance concerning Article 12(c), the Academy *recommends* that interested parties including relevant departments in the governments of the UK and the Academy work together to review and clarify the Guidance associated with Article12(c) to ensure proportionate interpretation of the Regulation and champion the role of qualified nutrition professionals in consumer communications. The Academy also *recommends* that the current situation whereby highly influential individuals (such as ‘celebrities’ and ‘social media influencers’ who lack nutrition science training) are outside of the scope of Article 12(c) in the Regulation is unacceptable and requires further consideration and action to ensure a level playing field.

## References

[1] Williams CM , Ashwell M , Prentice A , et al. (2021) Nature of the evidence base and frameworks underpinning dietary recommendations for prevention of non-communicable diseases: a position paper from the academy of nutrition sciences. Br J Nutr 126, 1076–1090. 34515022 10.1017/S0007114520005000

[2] European Union (2006) Regulation (EC) No 1924/2006 of the European parliament and of the council of 20 December 2006 on nutrition and health claims made on foods. Off J Eur Union 404, 3.

[3] Department of Health and Social Care (2021) Nutrition and Health Claims: Guidance to Compliance with Regulation (EC) 1924/2006. https://www.gov.uk/government/publications/nutrition-and-health-claims-guidance-to-compliance-with-regulation-ec-1924–2006-on-nutrition-and-health-claims-made-on-foods/nutrition-and-health-claims-guidance-to-compliance-with-regulation-ec-19242006 (accessed September 2022).

[4] Díaz LD , Fernández-Ruiz V & Cámara M (2020) An international regulatory review of food health-related claims in functional food products labeling. J Funct Foods 68, 103896.

[5] de Boer A (2021) Fifteen years of regulating nutrition and health claims in Europe: the past, the present and the future. Nutrients 13, 1725. 34069664 10.3390/nu13051725 PMC8161257

[6] Kusar A , Zmitek K , Lahteenmaki L , et al. (2021) Comparison of requirements for using health claims on foods in the European Union, the USA, Canada, and Australia/New Zealand. Compr Rev Food Sci Food Saf 20, 1307–1332. 33565710 10.1111/1541-4337.12716

[7] European Union (1990) EU 1990. Council directive 90/496/EEC of 24 September 1990 on nutrition labelling for foodstuffs. Off J 276, 40.

[8] European Union (2011) Regulation (EU) No 1169/2011 of the European Parliament and of the Council of 25 October 2011 on the Provision of Food Information to Consumers, Amending Regulations (EC) No 1924/2006 and (EC) No 1925/2006 of the European Parliament and of the Council, and Repealing Commission Directive 87/250/EEC, Council Directive 90/496/EEC, Commission Directive 1999/10/EC, Directive 2000/13/EC of the European Parliament and of the Council, Commission Directives 2002/67/EC and 2008/5/EC and Commission Regulation (EC) No 608/2004. https://www.legislation.gov.uk/eur/2011/1169/contents (accessed September 2022).

[9] Contor L (2001) Functional food science in Europe. Nutr Metab Cardiovasc Dis 11, 20–23. 11894747

[10] Aggett PJ , Antoine JM , Asp NG , et al. (2005) PASSCLAIM: consensus on criteria. Eur J Nutr 44, Suppl. 1, i5–i30. 15933809 10.1007/s00394-005-1104-3

[11] Department of Health and Social Care Guidance Nutrition and Health Claims: Guidance to Compliance with Regulation (EC) 1924/2006. https://www.gov.uk/government/publications/nutrition-and-health-claims-guidance-to-compliance-with-regulation-ec-1924–2006-on-nutrition-and-health-claims-made-on-foods/nutrition-and-health-claims-guidance-to-compliance-with-regulation-ec-19242006 (accessed September 2022).

[12] European Union EU Register of Health Claims (2021). https://ec.europa.eu/food/safety/labelling_nutrition/claims/register/public/?event=search (accessed March 2022).

[13] EFSA Panel on Dietetic Products Nutrition and Allergies (2021) General scientific guidance for stakeholders on health claim applications (Revision 1). EFSA J 19, 6553. 10.2903/j.efsa.2021.6553 PMC7996104 33791037

[14] Verhagen H , Vos E , Francl S , et al. (2010) Status of nutrition and health claims in Europe. Arch Biochem Biophys 501, 6–15. 20417175 10.1016/j.abb.2010.04.012

[15] Verhagen H & van Loveren H (2016) Status of nutrition and health claims in Europe by mid 2015. Trends in Food Science and Technology 56, 39–45.

[16] Ruffell M (2003) Health claims on food – JHCI update substantiation requirements. BNF Nutr Bull 28, 193–196.

[17] UK Nutrition and Health Claims Committee (2020). https://www.gov.uk/government/groups/uk-nutrition-and-health-claims-committee (accessed September 2022).

[18] European Union (2017) Commission Regulation (EU) No 432/2012 of 16 May 2012 Establishing a List of Permitted Health Claims Made on Foods, Other Than Those Referring to the Reduction of Disease Risk and to Children’s Development and Health. https://eur-lex.europa.eu/LexUriServ/LexUriServ.do?uri=OJ:L:2012:136:0001:0040:en:PDF (accessed October 2022).

[19] EFSA Panel on Dietetic Products Nutrition and Allergies (2015) Vitamin C and contribution to the normal function of the immune system: evaluation of a health claim pursuant to Article 14 of Regulation (EC) No 1924/2006 EFSA J 13, 4298–4305. 10.2903/j.efsa.2015.4298 PMC13189435 42169835

[20] EFSA Panel on Dietetic Products Nutrition and Allergies (2009) Scientific opinion on the substantiation of health claims related to calcium and vitamin D and maintenance of bone (ID 350) pursuant to Article 13(1) of Regulation (EC) No 1924/2006. EFSA J 7, 1272–1286.

[21] EFSA Panel on Dietetic Products Nutrition and Allergies (2011) Scientific opinion on the substantiation of health claims related to resistant starch and reduction of post-prandial glycaemic responses (ID 681), “digestive health benefits” (ID 682) and “favours a normal colon metabolism” (ID 783) pursuant to Article 13(1) of Regulation (EC) No 1924/20061. EFSA J 9, 2024. 10.2903/j.efsa.2011.2024 PMC13129663 42078732

[22] EFSA Panel on Dietetic Products Nutrition and Allergies (2011) Scientific opinion on the substantiation of health claims related to carbohydrate-electrolyte solutions and reduction in rated perceived exertion/effort during exercise (ID 460, 466, 467, 468), enhancement of water absorption during exercise (ID 314, 315, 316, 317, 319, 322, 325, 332, 408, 465, 473, 1168, 1574, 1593, 1618, 4302, 4309), and maintenance of endurance performance (ID 466, 469) pursuant to Article 13(1) of Regulation (EC) No 1924/2006. EFSA J 9, 2211.

[23] European Commission (2020) Commission Staff Working Document Evaluation of the Regulation (EC) No 1924/2006 on Nutrition and Health Claims Made on Foods with Regard to Nutrient Profiles and Health Claims Made on Plants and Their Preparations and of the General Regulatory Framework for Their Use in Foods. https://food.ec.europa.eu/system/files/2020-05/labelling_nutrition-claims_swd_2020-96_sum_en.pdf (accessed October 2022).

[24] EFSA Panel on Dietetic Products Nutrition and Allergies (2011) Scientific opinion on the substantiation of a health claim related to sugar beet fibre and increasing faecal bulk pursuant to Article 13(5) of Regulation (EC) No 1924/20061. EFSA J 9, 2468. 10.2903/j.efsa.2011.2468 PMC13093149 42015981

[25] EFSA Panel on Dietetic Products Nutrition and Allergies (2016) Creatine in combination with resistance training and improvement in muscle strength: evaluation of a health claim pursuant to Article 13(5) of Regulation (EC) No 1924/2006. EFSA J 14, 4400–4417. 10.2903/j.efsa.2016.4400 PMC13093162 42016888

[26] HM Government (2021) Medicines and Medical Devices Act. https://www.legislation.gov.uk/ukpga/2021/3/contents (accessed September 2022).

[27] EFSA Panel on Dietetic Products Nutrition and Allergies (2008) Scientific substantiation of a health claim related to plant stanol esters and lower/reduced blood cholesterol and reduced risk of (coronary) heart disease pursuant to Article 14 of Regulation (EC) No 1924/2006. EFSA J 825, 1–13.

[28] European Union (2013) Commission regulation (EU) No 718/2013 of 25 July 2013 amending regulation (EC) No 608/2004 concerning the labelling of foods and food ingredients with added phytosterols, phytosterol esters, phytostanols and/or phytostanol esters. Off J Eur Union L201, 49–50. https://eur-lex.europa.eu/legal-content/EN/TXT/PDF/?uri=CELEX:32013R0718&from=PL

[29] EFSA Panel on Dietetic Products Nutrition and Allergies (2011) Scientific opinion on the substantiation of a health claim related to “low fat and low trans spreadable fat rich in unsaturated and *n*-3 fatty acids” and reduction of LDL-cholesterol concentrations pursuant to Article 14 of Regulation (EC) No 1924/2006. EFSA J 9, 2168. 10.2903/j.efsa.2011.2168 PMC13177346 42148400

[30] Department of Health and Social Care (2022) Great Britain Nutrition and Health Claims (NHC) Register. https://www.gov.uk/government/publications/great-britain-nutrition-and-health-claims-nhc-register (accessed September 2022).

[31] EFSA Panel on Dietetic Products Nutrition and Allergies (2008) Scientific substantiation of a health claim related to calcium and bone growth pursuant to Article 14 of Regulation (EC) No 1924/2006. EFSA J 826, 1–11.

[32] EFSA Panel on Dietetic Products Nutrition and Allergies (2015) Scientific opinion on the substantiation of a health claim related to vitamin D and contribution to the normal function of the immune system pursuant to Article 14 of Regulation (EC) No 1924_2006. EFSA J 13, 4096. 10.2903/j.efsa.2014.3653 PMC13159920 42125363

[33] European Food Safety Authority (2022). https://www.efsa.europa.eu/en/topics/topic/health-claims#:~:text=EFSA%20is%20to%20verify%20that,manner%20for%20their%20specific%20products (accessed October 2022).

[34] Rob2 Development Group (2019) Revised Cochrane Risk-of-Bias Tool for Randomized Trials (RoB 2). https://sites.google.com/site/riskofbiastool/welcome/rob-2–0-tool/current-version-of-rob-2 (accessed September 2022).

[35] Williams CM , Lovegrove JA & Griffin BA (2013) Dietary patterns and cardiovascular disease. Proc Nutr Soc 72, 407–411. 23953031 10.1017/S0029665113002048

[36] Gibney MJ (2019) Ultra-processed foods: definitions and policy issues. Curr Dev Nutr 3, nzy077. 30820487 10.1093/cdn/nzy077 PMC6389637

[37] Mozaffarian D , El-Abbadi NH & O’Hearn M (2021) Food compass is a nutrient profiling system using expanded characteristics for assessing healthfulness of foods. Nature Food 2, 809–818. 37117986 10.1038/s43016-021-00381-y

[38] Department of Health and Social Care (2020) Nutrition Related Labelling, Composition and Standards Provisional Common Framework. https://assets.publishing.service.gov.uk/government/uploads/system/uploads/attachment_data/file/925713/Nutrition_related_labelling__composition_and_standards_provisional_common_framework__web_accessible_.pdf (accessed September 2022).

[39] EFSA Scientific Committee (2021) Guidance on uncertainty in EFSA scientific assessment. EFSA J 49, 219.

[40] EFSA Panel on Dietetic Products Nutrition and Allergies (2009) Scientific opinion on the substantiation of health claims related to non characterised microorganisms pursuant to Article 13(1) of Regulation (EC) No 1924/2006. EFSA J 7, 1247.

[41] EFSA Panel on Dietetic Products Nutrition and Allergies (2011) Scientific opinion on the substantiation of a health claim related to sugar beet fibre and decreasing intestinal transit time pursuant to Article 13(5) of Regulation (EC) No 1924/2006. EFSA J 9, 2467. 10.2903/j.efsa.2011.2467 PMC13093155 42015980

[42] EFSA Panel on Dietetic Products Nutrition and Allergies (2011) Scientific opinion on the substantiation of health claims related to arabinoxylan produced from wheat endosperm and reduction of post-prandial glycaemic responses (ID 830) pursuant to Article 13(1) of Regulation (EC) No 1924/2006. EFSA J 9, 2205. 10.2903/j.efsa.2011.2024 PMC13129663 42078732

[43] US Food and Drug Administration. Guidance for Industry: Evidence-Based Review System for the Scientific Evaluation of Health Claims. https://www.fda.gov/regulatory-information/search-fda-guidance-documents/guidance-industry-evidence-based-review-system-scientific-evaluation-health-claims (accessed October 2022).

[44] US Food and Drug Administration (2017) Questions and Answers on Health Claims in Food Labeling. https://www.fda.gov/food/food-labeling-nutrition/questions-and-answers-health-claims-food-labeling (accessed September 2022).

[45] US Food and Drug Administration (2018) Authorized Health Claims That Meet the Significant Scientific Agreement (SSA) Standard. https://www.fda.gov/food/food-labeling-nutrition/authorized-health-claims-meet-significant-scientific-agreement-ssa-standard (accessed September 2022).

[46] US Food and Drug Administration. https://www.fda.gov/food/food-labeling-nutrition/qualified-health-claims (accessed October 2022).

[47] US Food and Drug Administration (2019) Qualified Health Claims. https://www.todaysdietitian.com/newarchives/010614p10.shtml (accessed September 2022).

[48] US Food and Drug Administration. Title FDA Modernization Act (FDAMA) Claims. https://www.fda.gov/food/food-labeling-nutrition/fda-modernization-act-fdama-claims (accessed October 2022).

[49] Yamada K , Sato-Mito N , Nagata J , et al. (2008) Health claim evidence requirements in Japan. J Nutr 138, 1192S–1198S. 18492856 10.1093/jn/138.6.1192S

[50] Tapsell LC (2008) Evidence for health claims: a perspective from the Australia-New Zealand region. J Nutr 138, 1206S–1209S. 18492858 10.1093/jn/138.6.1206S

[51] Neale EP & Tapsell LC (2019) Perspective: the evidence-based framework in nutrition and dietetics: implementation, challenges, and future directions. Adv Nutr 10, 1–8. 30649173 10.1093/advances/nmy113 PMC6370257

[52] Neale EP & Tapsell LC (2022) Nutrition and health claims: consumer use and evolving regulation. Curr Nutr Rep 11, 431–436. 35606620 10.1007/s13668-022-00422-3 PMC9381452

[53] Food Standards Australia and New Zealand (2020) Nutrition, Health and Related Claims. https://www.foodstandards.gov.au/industry/labelling/Pages/Nutrition-health-and-related-claims.aspx (accessed September 2022).

[54] Food Standards Australia and New Zealand (2018) Getting Your Claims Right: A Guide to Complying with the Nutrition, Health and Related Claims Standard of the Australia New Zealand Food Standards Code. https://foodregulation.gov.au/internet/fr/publishing.nsf/Content/31BDC68CEC4A1964CA25801B00166C1F/$File/Getting-Your-Claims-Right-2018.pdf (accessed September 2022).

[55] Australian Government Food Standards Code (2015). https://www.legislation.gov.au/Details/F2017C00711 (accessed September 2022).

[56] Wellard-Cole L , Watson WL , Hughes C , et al. (2019) How effective is food industry self-substantiation of food-health relationships underpinning health claims on food labels in Australia? Public Health Nutr 22, 1686–1695. 30829196 10.1017/S1368980018004081 PMC10260819

[57] Australian Government Overview of Nutrient Profiling Scoring System. https://www.foodstandards.gov.au/industry/labelling/Pages/Consumer-guide-to-NPSC.aspx (accessed October 2022).

[58] Department of Health (2011) Nutrient Profiling Technical Guidance. https://assets.publishing.service.gov.uk/government/uploads/system/uploads/attachment_data/file/216094/dh_123492.pdf (accessed October 2022).

[59] Verhagen H , Vos E , Francl S , et al. (2010) Status of nutrition and health claims in Europe. Arch Biochem Biophys 501, 6–15. 20417175 10.1016/j.abb.2010.04.012

[60] European Union (2002) Regulation (EC) no 178/2002 of the European parliament and of the council of 28 January 2002 laying down the general principles and requirements of food law, establishing the European food safety authority and laying down procedures in matters of food safety. https://eur-lex.europa.eu/legal-content/EN/ALL/?uri=celex%3A32002R0178 (accessed October 2022).

[61] HM Government UK Nutrition and Health Claims Committee. https://www.gov.uk/government/groups/uk-nutrition-and-health-claims-committee (accessed October 2022).

[62] EFSA Panel on Dietetic Products Nutrition and Allergies (NDA) (2012) Scientific Opinion on the substantiation of a health claim related to glucose and contribution to energy‐yielding metabolism pursuant to Article 13(5) of Regulation (EC) No 1924/2006. EFSA J 10, 2694–2704. 10.2903/j.efsa.2012.2694 PMC13151560 42112180

[63] European Commission (2015) Commission Regulation (EU) 2015/8 of 6 January 2015 Refusing to Authorise Certain Health Claims Made on Foods, Other Than Those Referring to the Reduction of Disease Risk and to Children’s Development and Health. https://eur-lex.europa.eu/legal-content/EN/TXT/PDF/?uri=CELEX:32015R0008&from=FR (accessed October 2022).

[64] EFSA Panel on Dietetic Products Nutrition and Allergies (2014) Scientific opinion on the substantiation of a health claim related to caffeine and increased alertness pursuant to Article 13(5) of Regulation (EC) No 1924/2006. EFSA J 2014, 3574–3590.

[65] European Food Safety Authority (2021) Nutrition Applications: Regulations and Guidance. https://www.efsa.europa.eu/en/applications/nutrition/regulationsandguidance (accessed September 2022).

[66] Witkamp RF (2021) Nutrition to optimise human health-how to obtain physiological substantiation? Nutrients 13, 2155. 34201670 10.3390/nu13072155 PMC8308379

[67] Wagner K-H , Cameron-Smith D , Wessner B , et al. (2016) Biomarkers of ageing; from function to molecular biology. Nutrients 8, 338. 27271660 10.3390/nu8060338 PMC4924179

[68] Levine M , Lu A , Quach A , et al. (2018) An epigenetic marker of ageing for lifespan and healthspan. Aging 10, 573–591. 29676998 10.18632/aging.101414 PMC5940111

[69] Joost HG , Gibney MJ , Cashman KD , et al. (2007) Personalised nutrition: status and perspectives. Br J Nutr 98, 26–31. 17381877 10.1017/S0007114507685195

[70] Williams CM , Ordovas JM , Lairon D , et al. (2008) The challenges for molecular nutrition research 1: linking genotype to healthy nutrition. Genes Nutr 3, 41–49. 18850186 10.1007/s12263-008-0086-1 PMC2467452

[71] van Ommen B , Keijer J , Kleemann R , et al. (2008) The challenges for molecular nutrition research 2: quantification of the nutritional phenotype. Genes Nutr 3, 51–59. 18850187 10.1007/s12263-008-0084-3 PMC2467450

[72] van Ommen B , Fairweather-Tait S , Freidig A , et al. (2008) A network biology model of micronutrient related health. Br J Nutr 99, Suppl. 3, S72–S80. 18598592 10.1017/S0007114508006922

[73] Public Health England (2020) Adult Obesity Prevalence by National Statistics Socio-Economic Classification. https://www.gov.uk/government/publications/adult-obesity-prevalence-by-national-statistics-socio-economic-classification (accessed October 2022).

[74] Public Health England (2017) Hypertension prevalence estimates in England, 2017. Estimated from the Health Survey for England. https://assets.publishing.service.gov.uk/government/uploads/system/uploads/attachment_data/file/873605/Summary_of_hypertension_prevalence_estimates_in_England__1_.pdf (accessed October 2022).

[75] NHS Digital (2019) Health Survey for England. https://digital.nhs.uk/data-and-information/publications/statistical/health-survey-for-england (accessed October 2022).

[76] Kardinaal AF , van Erk MJ , Dutman AE , et al. (2015) Quantifying phenotypic flexibility as the response to a high-fat challenge test in different states of metabolic health. FASEB J 29, 4600–4613. 26198450 10.1096/fj.14-269852

[77] World Health Organisation (2019) WHO Guiding Principles and Framework Manual for Front-of-Pack Labelling for Promoting Healthy Diet. https://cdn.who.int/media/docs/default-source/healthy-diet/guidingprinciples-labelling-promoting-healthydiet.pdf?sfvrsn=65e3a8c1_7&download=true (accessed October 2022).

[78] European Food Safety Authority The Science Behind Nutrient Profiling – Have Your Say. https://www.efsa.europa.eu/en/news/science-behind-nutrient-profiling-have-your-say (accessed September 2022).

[79] Williams P (2005) Consumer understanding and use of health claims for foods. Nutr Rev 63, 256–264. 16121480 10.1111/j.1753-4887.2005.tb00382.x

[80] Kaur A , Scarborough P & Rayner M (2017) A systematic review, and meta-analyses, of the impact of health-related claims on dietary choices. Int J Behav Nutr Phys Act 14, 93. 28697787 10.1186/s12966-017-0548-1 PMC5505045

[81] Mohebalian PM , Cernusca MM & Aguilar FX (2012) Discovering Niche markets for elderberry juice in the United States. HortTechnology 22, 556.

[82] Hieke S & Grunert KG (2018) Consumers and health claims. In Foods, Nutrients and Food Ingredients with Authorised EU Health Claims, pp. 19–32 [MJ Sadler, editor]. Elsevier.

[83] Hung Y & Verbeke W (2019) Consumer evaluation, use and health relevance of health claims in the European Union. Food Qual Prefer 74, 88–99.

[84] Hodgkins CE , Egan B , Peacock M , et al. (2019) Understanding how consumers categorise health related claims on foods: a consumer-derived typology of health-related claims. Nutrients 11, 539. 30832373 10.3390/nu11030539 PMC6471133

[85] Bilman EM , Kleef Ev , Mela DJ , et al. (2012) Consumer understanding, interpretation and perceived levels of personal responsibility in relation to satiety-related claims. Appetite 59, 912–920. 22841815 10.1016/j.appet.2012.07.010

[86] Lähteenmäki L , Lampila P , Grunert K , et al. (2010) Impact of health-related claims on the perception of other product attributes. Food Policy 35, 230–239.

[87] Orquin JL & Scholderer J (2015) Consumer judgments of explicit and implied health claims on foods: misguided but not misled. Food Policy 51, 144–157.

[88] Shepherd R , Dean M , Lampila P , et al. (2012) Communicating the benefits of wholegrain and functional grain products to European consumers. Trends Food Sci Technol 25, 63–69.

[89] Hooker NH & Teratanavat R (2008) Dissecting qualified health claims: evidence from experimental studies. Crit Rev Food Sci Nutr 48, 160–176. 18274970 10.1080/10408390601177704

[90] Food and Drug Administration (2009) Experimental Study of Qualified Health Claims: Consumer Inferences about Monounsaturated Fatty Acids from Olive Oil, EPA and DHA *n*-3 Fatty Acids, and Green Tea. https://www.fda.gov/food/food-labeling-nutrition/experimental-study-qualified-health-claims-consumer-inferences-about-monounsaturated-fatty-acids-4.

[91] Buttriss J (2015) Nutrition and health claims in practice. Nutr Bull 40, 211–222.

[92] Bröring S , Khedkar S & Ciliberti S (2017) Reviewing the nutrition and health claims regulation (EC) no. 1924/2006: what do we know about its challenges and potential impact on innovation? Int J Food Sci Nutr 68, 1–9. 27484163 10.1080/09637486.2016.1212816

[93] Khedkar S , Bröring S & Ciliberti S (2017) Exploring the nutrition and health claims regulation (EC) no. 1924/2006: what is the impact on innovation in the EU food sector? Int J Food Sci Nutr 68, 10–17. 27484471 10.1080/09637486.2016.1212818

[94] Lockyer S , Ryder C , Jaworska S , et al. (2020) Developing a digital toolkit to enhance the communication of health claims: the health claims unpacked project. Nutr Bull 45, 432–443.

[95] Hieke S , Cascanette T , Pravst I , et al. (2016) The role of health-related claims and symbols in consumer behaviour: the CLYMBOL project. Agro Food Ind Hi-Tech 27, 26–29.

[96] Department of Health and Social Care (2013) Update on Flexibility of Wording for Health Claims. https://www.gov.uk/government/publications/update-on-flexibility-of-wording-for-health-claims (accessed October 2020).

[97] Advertising Standards Authority (2015) Food: Health Claims. https://www.asa.org.uk/advice-online/food-health-claims.html (accessed October 2020).

[98] de Boer A , Urlings M , Vos E , et al. (2015) Enforcement of the nutrition and health claim regulation. Eur Food Feed Law Rev 10, 334–344.

[99] de la Hunty A , Ashwell M , Arens U , et al. (2014) Authorised health claims may not help consumers to choose a healthy diet. Ann Nutr Metab 64, 1–5. 24642530 10.1159/000356127

[100] Talati Z , Pettigrew S , Neal B , et al. (2017) Consumers’ responses to health claims in the context of other on-pack nutrition information: a systematic review. Nutr Rev 75, 260–273. 28371913 10.1093/nutrit/nuw070

[101] Bröring S & Khedkar S (2018) Regulatory compliance and company strategies: the case of the nutrition and health claims regulation (EC) no. 1924/2006. In Regulating and Managing Food Safety in the EU: A Legal-Economic Perspective, pp. 105–128 [ H Bremmers and K Purnhagen , editors]. Cham: Springer International Publishing.

[102] Van Buul VJ & Brouns FJPH (2015) Nutrition and health claims as marketing tools. Crit Rev Food Sci Nutr 55, 1552–1560. 24364816 10.1080/10408398.2012.754738

[103] Grunert KG , Scholderer J & Rogeaux M (2011) Determinants of consumer understanding of health claims. Appetite 56, 269–277. 21238525 10.1016/j.appet.2011.01.009

[104] Tønnesen MT , Hansen S , Laasholdt A , et al. (2022) The impact of positive and reduction health claims on consumers’ food choices. Food Qual Prefer 98, 104526.

[105] Ruxton C & Ashwell M Dietitians’ and nutritionists’ knowledge and views on aspects of nutrition and health claims regulation in the UK: do we inadvertently shoot the messenger? BNF Nutr Bull. 10.1111/nbu.12616 37070365

